# Does country of resettlement influence the risk of labor market marginalization among refugees? A cohort study in Sweden and Norway

**DOI:** 10.5271/sjweh.4154

**Published:** 2024-05-01

**Authors:** Ridwanul Amin, Ellenor Mittendorfer-Rutz, Karina Undem, Ingrid Sivesind Mehlum, Rachel Louise Hasting

**Affiliations:** 1 Karolinska Institutet, Department of Clinical Neuroscience, Division of Insurance Medicine, Stockholm, Sweden.; 2 National Institute of Occupational Health, Oslo, Norway.; 3 Institute of Health and Society, University of Oslo, Oslo, Norway.; 4 Department of Occupational Medicine, Copenhagen University Hospital - Bispebjerg, Copenhagen, Denmark.; 5 Institute of Public Health, Copenhagen University, Copenhagen, Denmark.

**Keywords:** disability pension, length of stay, longitudinal, sick leave, unemployment

## Abstract

**Objectives:**

This study aimed to compare the risk of labor market marginalization among refugees across different host countries of resettlement and examine the moderating role of birth country and length of stay on these associations.

**Methods:**

Cohorts of refugees and native-born individuals aged 19–60 in Sweden (N=3 605 949, 3.5% refugees) and Norway (N=1 784 861, 1.7% refugees) were followed during 2010–2016. Rates (per 1000 person-years) of long-term unemployment, long-term sickness absence, and disability pension were estimated for refugees and the host populations. Cox regression models estimated crude and adjusted (for sex, age, educational level, and civil status) hazard ratio (HR_adj_) for refugees compared to their respective host population, with 95% confidence intervals (CI). Analyses were also stratified by birth country and length of stay.

**Results:**

Refugees in Norway and Sweden had a higher incidence of labor market marginalization compared to their host population. Refugees in Sweden had a comparatively lower relative risk of long-term unemployment but higher risk of disability pension (HR_adj_ 3.44, 95% CI, 3.38–3.50 and HR_adj_ 2.45, 2.35–2.56, respectively) than refugees in Norway (HR_adj_ 3.70, 3.58–3.82 and HR_adj_ 1.57, 1.49–1.66, respectively). These relative risks varied when stratifying by birth country. A shorter length of stay was associated with a higher risk of long-term unemployment and a lower risk of disability pension, with a stronger gradient in Sweden than in Norway.

**Conclusions:**

The relative risk of labor market marginalization varied by the refugees’ birth country but followed similar trends in Sweden and Norway. Although speculative, these findings may hint at non-structural factors related to the refugee experience playing a more important role than host country structural factors for the risk of labor market marginalization among refugees. Future research, including host countries with more variability in structural factors, is required to further investigate these associations. The higher risk of long-term unemployment among refugees with shorter length of stay indicates a need for more efficient labor market integration policies for newly-arrived refugees.

The unprecedented increase in forcible displacements around the world since the 1980s has resulted in an increasing number and diversity of resettled refugees in many European countries, including Sweden and Norway (1, 2). Between 2004 and 2021, around 353 000 and 110 000 refugees resettled in Sweden and Norway, respectively (3, 4). In 2020, 6.5 and 4.4% of the population of Sweden and Norway, respectively, were immigrants with a refugee background (5).

Most, if not all, individuals with a refugee background have experienced one or more traumatic events prior to or during migration, which increases the likelihood of mental ill-health (1, 6, 7). Upon arrival, refugees also have a higher prevalence of somatic disorders compared to the host population (8). Additionally, lower socioeconomic status and the social difficulties associated with forced displacement negatively affect health and work ability among refugees (9–11). A combination of these factors may lead to an increased vulnerability among refugees to find and sustain jobs (12), potentially leading to labor market marginalization (13).

Labor market marginalization is described as “the difficulty to obtain and keep a job” and is considered an economic as well as a public health problem (14). Prior evidence suggests that refugees face greater challenges integrating into the labor market, compared with the host population (13, 15, 16). In addition to unemployment, medical-based measures such as sickness absence and disability pension can be conceptualized as labor market marginalization. Refugees also have differing patterns of these benefits compared with their host population, among other reasons, due to the abovementioned health issues (13, 16). Additionally, difficulties in finding and keeping a job negatively affect refugees’ mental and somatic health, thereby inducing a vicious spiral of further labor market marginalization (17–19).

Several non-structural factors related to the heterogeneity among refugees seem to predict patterns of labor market marginalization (16, 19), such as socioeconomic status, health condition, language skills post resettlement (20), and traumatic experiences faced prior to and during migration (6). Structural factors in the host country, including social insurance policies, healthcare systems, migration policies, and unemployment rates, may further affect refugees’ integration and risk of labor market marginalization (5). Comparing the risk of labor market marginalization among refugees from the same birth country who resettled in different host countries will, to some extent, account for the within-group heterogeneity among refugees and may provide insight into how host-country-specific structural factors influence labor market marginalization risk. To our knowledge, no previous study has explored such potential effects of intercountry differences in structural factors on the subsequent labor market marginalization risk among refugees.

While Sweden and Norway share similarities in governance, welfare system and labor market structure, there are notable differences that may impact labor market marginalization risk. Firstly, regulations regarding social welfare benefits for labor market marginalization are less strict in Norway (16, 21, 22). Secondly, since 2010, yearly unemployment rates in Sweden have been almost twice that in Norway (23). Although both countries historically adopted similar integration policies (24), newly arrived refugees seem to be better integrated into the labor market in Norway than Sweden (25). These factors suggest that, compared to the respective host population, resettled refugees face a relatively lower labor market marginalization risk in Norway than Sweden.

Birth country can play a crucial role in refugees’ labor market marginalization due to differences in factors like validation of educational qualifications, proficiency in host country language, and discrimination (13, 26). Moreover, labor market integration seems to improve with increasing length of stay in the host country (27, 28). The similarities and differences in structural factors across host countries may also moderate the effect of length of stay on labor market marginalization risk, underscoring the importance of considering length of stay when comparing different host countries.

Our study aims to compare the risk of labor market marginalization among refugees across different host countries of resettlement. For this purpose, we estimated the risk of three labor market marginalization outcomes – long-term unemployment, long-term sickness absence, and disability pension – among refugees resettled in two different host countries, namely Sweden and Norway. We estimated the absolute risk of these outcomes among refugees as well as the relative risk compared to their respective host-country majority population – Swedish-and Norwegian-born individuals. Additionally, we aimed to examine the moderating role of birth country and length of stay on these associations.

## Methods

### Design and study population

In this nationwide cohort study in Sweden and Norway, the initial study population comprised all individuals aged 19–60 years on 31 December 2009 (baseline) and residents in the respective host country on 31 December 2007, 2008, and 2009; N=4 949 051 in Sweden and 2 475 274 in Norway. Exclusions were made for individuals with uncertain reason for immigration, and therefore, undeterminable migration status (N=163 123 and 101 562), individuals without gainful employment (ie, registered as employed and receiving a salary) in November 2009 (N=1 097 785 and 450 867), individuals with ongoing disability pension on 1 January 2010 (N=82 194 and 47 339), and non-refugee immigrants, as we focused on refugees as the study population (N=257 337 and 90 645). The final study populations of 3 348 612 and 1 784 861 individuals in Sweden and Norway, respectively, were followed from 1 January 2010 until 31 December 2016. The supplementary material (www.sjweh.fi/article/4154) figure S1 illustrates these selection steps.

### Data sources

Data on the following variables were obtained through registry linkages. Registries in Sweden included (i) *longitudinal integration database for labor market studies* (29): age, sex, birth country, educational level, civil status, area of residence, unemployment, sickness absence, disability pension, year of immigration, and involvement in the labor market; *(ii) longitudinal database for integration studies* (30): reason for immigration, length of stay, age at arrival; (iii) *micro data for analysis of the social insurance* database: date of being granted disability pension. Registries in Norway included (i) *Statistics Norway* (31, 32): age, sex, birth country, area of residence, civil status, reason for immigration, length of stay and educational level; and (ii) *Statistics Norway´s events database “FD-Trygd”* (33): start and end date of unemployment, sickness absence, disability pension, and employment-related information. Other registries in both countries comprised (i) *national patient registries* in Sweden (34, 35) and Norway (36): month and cause of psychiatric and somatic inpatient and specialized outpatient healthcare; and (ii) *Cause of Death* registries in Sweden (37) and Norway (38): date of death.

### Exposure measures

The exposure of interest was migration status. Those born in the host population – Swedish-born and Norwegian-born – were considered as the comparison group. Any individual with “refugee” listed as a reason for immigration to Sweden or Norway was identified as a refugee. Refugees were further categorized according to their birth country (Eritrea, Ethiopia, Somalia, other countries in Africa, Afghanistan, Iran, Iraq, Syria, other countries in Asia, Chile, other countries in South America, former Yugoslavian countries, and other countries outside Africa, Asia and South America) (13), as well as their length of stay (2–5, 6–10, and >10 years) (28, 39) in the host country.

### Outcome measures

To facilitate comparability with previous studies (13, 28, 39), the outcome measures were long-term unemployment (>180 annual unemployment days), long-term sickness absence (>90 days per spell; full or partial), and receipt of disability pension (full or partial). All variables were coded as yes/no, and the first day of the respective outcome was identified for the time-to-event analysis. For long-term unemployment, the first day of the calendar year with >180 days of unemployment was used.

Resettled refugees in Sweden and Norway are entitled to the same social welfare benefits as their native-born counterparts. A summary of the social insurance systems in the host countries is provided in the supplementary material.

### Covariates

Covariates included *socio-demographic factors (measured at baseline)*: age, sex, educational level, civil status, area of residence; *labor market marginalization factors*: history of unemployment, sickness absence, and disability pension; and *healthcare factors*: any inpatient and specialized outpatient healthcare due to mental or somatic disorders, measured in the two years preceding follow-up (ie, 2008–2009).

Table 1 shows the categories of the covariates and the International Classification of Diseases version-10 (ICD-10) codes used for the healthcare factors. Missing values for a covariate were coded as a separate category.

**Table 1:** Descriptive statistics of socio-demographic, labor market marginalization, morbidity and migration-related factors of all gainfully employed individuals with a refugee or host population background, aged 19–60 years on 31 December 2009, and resident in the respective host country on 31 December 2007, 2008, and 2009 (N=3 348 612 and 1 784 861 in Sweden and Norway, respectively). [NA=not available.]

Characteristics		Swedish-born		Refugees in Sweden		Norwegian-born		Refugees in Norway
		N (column %)		N (column %)		N (column %)		N (column %)
All (row %)		3 230 343 (96.5)		118 269 (3.5)		1 754 240 (98.3)		30 621 (1.7)
Sex								
	Women	1 544 342 (47.8)		47 377 (40.1)		833 967 (47.5)		11 543 (37.7)
	Men	1 686 001 (52.2)		70 892 (59.9)		920 273 (52.5)		19 078 (62.3)
Age (years)								
	19–24	335 998 (10.4)		11 737 (9.9)		218 478 (12.5)		4 604 (15.0)
	25–34	708 547 (21.9)		28 642 (24.2)		386 560 (22.0)		9 199 (30.0)
	35–44	913 369 (28.3)		34 310 (29.0)		484 440 (27.6)		9 979 (32.6)
	45–54	823 087 (25.5)		35 374 (29.9)		442 934 (25.3)		5 733 (18.7)
	55–60	449 342 (13.9)		8 206 (6.9)		221 828 (12.7)		1106 (3.6)
Educational level (years)								
	Elementary (0–9)	306 885 (9.5)		21 001 (17.8)		277 618 (15.8)		10 110 (33.0)
	High school (10–12)	1 670 565 (51.7)		55 928 (47.3)		809 756 (46.2)		10 408 (34.0)
	University/college (>12)	1 250 440 (38.7)		40 486 (34.2)		665 211 (37.9)		8 844 (28.9)
	Missing information	2453 (0.1)		854 (0.7)		1655 (0.1)		1259 (4.1)
Civil status								
	Single	1 900 798 (58.8)		48 997 (41.4)		977 520 (55.7)		12 970 (42.4)
	Married/civil partnership	1 329 545 (41.2)		69 272 (58.6)		776 720 (44.3)		17 651 (57.6)
Type of living area								
	Large cities ^a^	1 189 955 (36.8)		67 422 (57.0)		829 367 (47.3)		18 569 (60.6)
	Outside large cities	3 230 343 (63.2)		118 269 (43.0)		924 873 (52.7)		12 052 (39.4)
	Sickness absence in 2009 (Yes) ^b^	265 758 (8.2)		13 067 (11.0)		27 746 (4.2)		845 (5.9)
Unemployment in 2009 (days) ^b^								
	1–180	205 652 (6.4)		14 350 (12.1)		13 399 (0.8)		1 347 (4.4)
	>180	28 419 (0.9)		3106 (2.6)		2006 (0.1)		241 (0.8)
Somatic disorders in 2008–2009 (yes) ^b, c^		583 835 (18.1)		24 346 (20.6)		330 607 (18.9)		6236 (20.4)
Mental disorders in 2008–2009 (yes) ^b, d^		85 491 (2.6)		3981 (3.4)		44 973 (2.6)		1082 (3.5)
Length of stay in host country (years)								
	2–5	NA ^e^		8919 (7.5)		NA ^e^		5454 (17.8)
	6–10	NA ^e^		12 260 (10.4)		NA ^e^		10 976 (35.8)
	>10	NA ^e^		97 090 (82.1)		NA ^e^		14 191 (46.3)
Age at arrival in host country (years)								
	0–6	NA ^e^		7375 (6.2)		NA ^e^		1455 (4.8)
	7–13	NA ^e^		16 025 (13.6)		NA ^e^		3257 (10.6)
	14–16	NA ^e^		6777 (5.7)		NA ^e^		2051 (6.7)
	17–18	NA ^e^		5055 (4.3)		NA ^e^		1025 (3.4)
	>18	NA ^e^		83 037 (70.2)		NA ^e^		22 833 (74.6)

^a^ Stockholm, Gothenburg and Malmö in Sweden and Oslo, Stavanger, Trondheim, and Bergen in Norway. ^b^ ‘No unemployment’, ‘No sickness absence’, ‘No somatic disorder’ and, ‘No mental disorders’ categories are not presented. ^c^ Measured as any inpatient or specialized outpatient healthcare due to any somatic disorders (diabetes mellitus: International classification of diseases version 10 or ICD-10 codes E11, E14, E66); diseases of the nervous system: ICD-10 G20, G21, G30, G35, G43, G44, G47, G56, G62, G92; diseases of the circulatory system: ICD-10 I10-I15, I20-I25, I73, I83; diseases of the respiratory system: ICD-10 J30, J31, J40-J47, J60-J70, J84, J92; diseases of the musculoskeletal system: ICD-10 M16, M17, M50-M54, M65-M68, M70-M79; diseases of the ear and mastoid process: ICD-10 H80-H95; diseases of the skin and subcutaneous tissue: ICD-10: L23-L25, L40, L50; injury, poisoning and certain other consequences of external causes: ICD-10 S00-T98). ^d^ Measured as any inpatient or specialized outpatient healthcare due to any mental disorders (ICD-10 codes F00-F99). ^e^ Not applicable for host population.

### Statistical analyses

Separate analyses were performed for Sweden and Norway. Number of events per 1000 person-years (absolute rates) were calculated for each outcome, stratified by birth country and length of stay. In the multivariate-adjusted analyses, we adjusted for sex, age, educational level and civil status, after considering all covariates in a directed acyclic graph (DAG) (supplementary figure S2). Cox regression models yielding crude and multivariate-adjusted hazard ratios (HR and HR_adj_, respectively) with 95% confidence intervals (CI) were used to estimate the relative risk of labor market marginalization among refugees versus respective host population in Sweden and Norway. The assumption of proportional hazards was confirmed by plotting log-minus-log Kaplan–Meier survival curves and Schoenfeld residuals. Emigration, death, and end of follow-up, whichever occurred first, were censoring events. For the long-term unemployment and long-term sickness absence analyses, the receipt of disability pension was an additional censoring event. Individuals with ongoing sickness absence on 1 January 2010 were excluded from the analysis for long-term sickness absence (N=75 028 and 93 919 in Sweden and Norway, respectively). To test if length of stay and birth country interacts, we investigated the risk of labor market marginalization for a specific birth country (Eritrea, Ethiopia, Somalia, Afghanistan, Iraq, Iran, Syria, Chile and former Yugoslavia) and each stratum of length of stay (2–5 versus 6–10 versus >10 years). We used SAS 9.4 (SAS Institute, Cary, NC, USA) and Stata 16.1 (StataCorp, College Station, TX, USA) for the analyses in Sweden and Norway, respectively.

### Sensitivity analyses

A sensitivity analysis excluding the stratum missing educational level yielded consistent results with the main analysis.

## Results

In both Sweden and Norway, refugees were less likely to be women, aged 55–60 years, single, and living outside large cities, compared with the host population (table 1). The proportion of refugees with 0–9 years of education was almost double the proportion among Swedish-born (17.8% versus 9.5%) and Norwegian-born (33% versus 15.8%). A higher proportion of refugees in both countries had any sickness absence or unemployment benefits in 2009 than the host population. A much higher proportion of refugees in Sweden had lived there for >10 years (82.1%) compared to refugees in Norway (46.3%).

### Long-term unemployment among refugees

**Figure 1:**
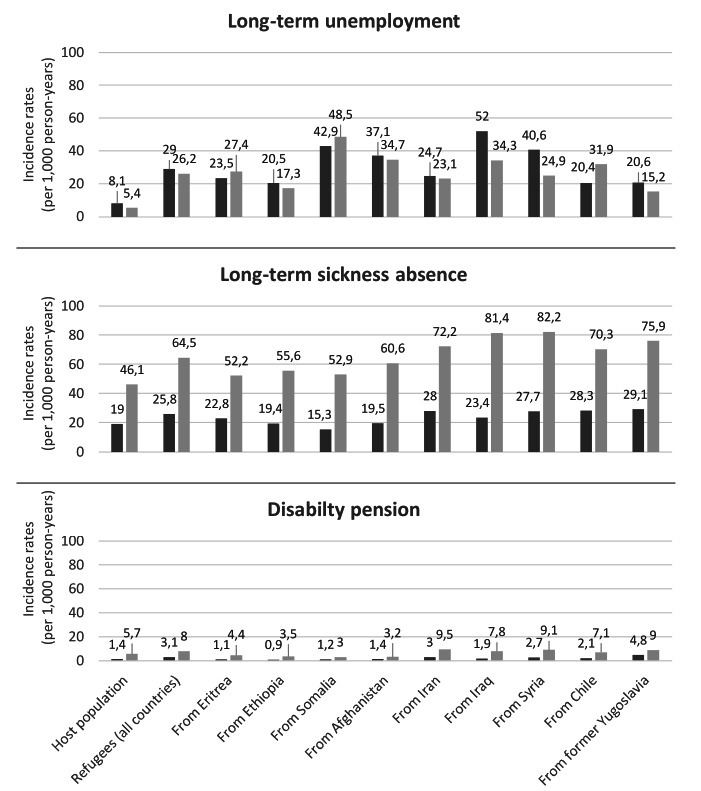
Incidence rates (per 1000 person-years) of long-term unemployment, long-term sickness absence and disability pension from 2010–2016 among gainfully employed refugees from specific countries^a^ of birth who resettled in Sweden and Norway and the host population (Swedish-born and Norwegian-born, respectively). ^a^ Countries which generated the largest number of refugees to Sweden and Norway.

The incidence of long-term unemployment among refugees in Sweden was 3.5 times higher than that among the Swedish-born (29/1000 person-years (1000PY) versus 8.1/1000PY), with some variations by their specific birth country (figure 1). Refugees from Iraq (52/1000PY) had more than 6 times the incidence of long-term unemployment among the Swedish-born. Similar differences in the incidence rates of long-term unemployment were found in Norway. Refugees had 4.8 times higher incidence of long-term unemployment than that of Norwegian-born (26.2/1000PY versus 5.4/1000PY). Incidence of long-term unemployment was lower in Norway than Sweden, both for the host populations and most refugee groups, except for refugees from Eritrea, Somalia, and Chile in Norway with higher rates than that of the same refugee groups in Sweden.

**Figure 2:**
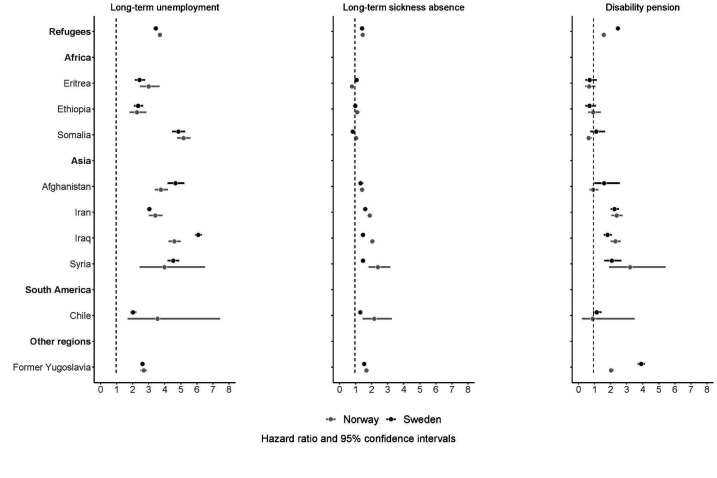
Risk of long-term unemployment, long-term sickness absence and disability pension from 2010–2016 among gainfully employed refugees from specific countriesa of birth who resettled in Sweden and Norway, compared with the Swedish-born and Norwegian-born population, respectively; multivariate-adjusted^b^ hazard ratios (aHRs) with 95% confidence intervals. ^a^ Countries which generated the largest number of refugees to Sweden and Norway.

The adjusted relative risk of long-term unemployment (refugees versus host population) during follow-up was comparatively higher among the refugees in Norway than those in Sweden (HR_adj_ 3.70, 95% CI 3.58–3.82 versus 3.44, 3.38–3.50; figure 2, Supplementary table S1). Although most refugee groups had a similar relative risk irrespective of host country of resettlement, refugees from Afghanistan and Iraq in Norway had a comparatively lower risk than refugees from the same countries who resettled in Sweden (figure 2).

### Long-term sickness absence among refugees

Refugee groups in both Sweden and Norway had higher rates of long-term sickness absence than their host population (figure 1). Only refugees from Somalia in Sweden had lower rates of long-term sickness absence (15.3/1000PY) than their Swedish-born counterparts (19/1000PY). Both the Norwegian-born host population and the country-specific refugee groups in Norway had 2–3 times higher incidence of long-term sickness absence than the corresponding groups in Sweden.

Refugees from African countries in both Sweden and Norway generally had no or little difference in adjusted risk of long-term sickness absence compared to their host populations (figure 2 and supplementary table S2). All other refugee groups had a higher risk of long-term sickness absence than the host populations. Refugees from a specific birth country resettling in Sweden generally had a comparatively lower relative risk of long-term sickness absence than those resettling in Norway.

### Disability pension among refugees

In Sweden, the incidence of disability pension among refugees was 2.2 times higher than that of the Swedish-born (3.1/1000PY versus 1.4/1000PY), while in Norway, it was 1.3 times higher (7.5/1000PY versus 5.7/1000PY). Refugees from African countries had lower rates of disability pension than the host population in Sweden (figure 1). On the other hand, refugees from former Yugoslavia (4.8/1000PY) and Iran (3/1000PY) in Sweden had a higher rate of disability pension. Lower rates of disability pension were also observed for the African refugee groups in Norway, along with refugees from Afghanistan (3.1/1000PY), compared to the Norwegian-born. Comparison between the host countries showed that the disability pension rate was 4 times higher among the Norwegian-born than the Swedish-born, and all refugee groups in Norway had higher rates of disability pension than the refugees from the same country who resettled in Sweden.

The adjusted risk of disability pension was higher for refugees compared to their host population, but relatively lower for refugees in Norway (HR_adj_ 1.57, 95% CI 1.49–1.66), compared to refugees in Sweden (HR_adj_ 2.45, 2.35–2.56) (supplementary table S3). Refugees from former Yugoslavia had a higher relative risk of disability pension in Sweden (HR_adj_ 3.90, 3.69–4.12) than in Norway (HR_adj_ 2.02, 1.87–2.18); refugees from all other birth countries had a similar relative risk of disability pension across the host countries (figure 2).

### Length of stay and labor market marginalization among refugees

Length of stay had a strong influence on the long-term unemployment risk among refugees in both host countries, with a comparatively stronger influence in Sweden than Norway (table 2). In Sweden, the difference in relative risk was almost twice that of Norway for those with 2–5 years of residency (HR_adj_ 8.69, 95% CI 8.36–9.04 versus HR_adj_ 4.46, 4.19–4.76). However, long-term unemployment relative risk among refugees with >10 years of residence was similar in Sweden and Norway (HR_adj_ 2.89, 2.83–2.95 versus HR_adj_ 2.76, 2.61–2.93),

**Table 2:** Risk of long-term unemployment, long-term sickness absence and disability pension from 2010–2016 among gainfully employed refugees compared to the Swedish-born and Norwegian-born population, respectively, stratified by their length of stay in Sweden and Norway; crude and multivariate hazard ratios (HR) with 95% confidence intervals (CI).

Length of stay			N events (%)	Rate/1000 person-years	Crude HR (CI)	Adjusted ^a^ HR (CI)
**Long-term unemployment**						
	Swedish-born		133 073 (4.1)	8.1	1	1
	Refugees in Sweden (years)					
		2–5	2697 (30.2)	73.6	8.25 (7.94–8.57)	8.69 (8.36–9.04)
		6–10	2257 (18.4)	42.3	4.89 (4.69–5.10)	5.30 (5.08–5.53)
		>10	10 674 (11.0)	23.7	2.82 (2.76–2.87)	2.89 (2.83–2.95)
	Norwegian-born		52 807 (3.0)	5.4	1	1
	Refugees in Sweden (years)					
		2–5	1075 (19.7)	39.4	7.01 (6.60–7.45)	4.46 (4.19–4.76)
		6–10	1683 (15.3)	32.2	5.71 (5.44–5.99)	4.31 (4.10–4.53)
		>10	1219 (8.6)	16.8	3.03 (2.86–3.20)	2.76 (2.61–2.93)
**Long-term sickness absence**						
	Swedish-born		367 216 (11.6)	19.0	1	1
	Refugees in Sweden (years)					
		2–5	1051 (12.0)	18.7	0.98 (0.92–1.04)	1.15 (1.08–1.22)
		6–10	1888 (15.9)	25.4	1.33 (1.28–1.40)	1.47 (1.40–1.54)
		>10	15 246 (16.2)	26.5	1.39 (1.37–1.41)	1.41 (1.38–1.43)
	Norwegian-born		357 008 (21.5)	37.7	1	1
	Refugees in Sweden (years)					
		2–5	1399 (27.0)	45.9	1.23 (1.17–1.30)	1.09 (1.03–1.15)
		6–10	3317 (33.2)	60.7	1.62 (1.57–1.68)	1.60 (1.55–1.66)
		>10	3995 (30.5)	55.4	1.47 (1.43–1.52)	1.45 (1.40–1.49)
**Disability pension**						
	Swedish-born		28 021 (0.9)	1.4	1	1
	Refugees in Sweden (years)					
		2–5	89 (1.0)	1.5	1.08 (0.88–1.33)	1.65 (1.34–2.04)
		6–10	229 (1.9)	2.8	2.05 (1.80–2.33)	2.66 (2.34–3.03)
		>10	2131 (2.2)	3.3	2.46 (2.35–2.57)	2.47 (2.36–2.58)
	Norwegian-born		64 095 (3.7)	5.7	1	1
	Refugees in Sweden (years)					
		2–5	134 (2.5)	3.6	0.63 (0.53–0.74)	0.79 (0.67–0.94)
		6–10	544 (5.0)	7.3	1.62 (1.57–1.68)	1.60 (1.55–1.66)
		>10	872 (6.1)	9.2	1.63 (1.52–1.74)	1.73 (1.62–1.85)

^a^ Adjusted for sex, age, educational level and civil status.

Refugees’ length of stay did not seem to have any modifying effect on risk of long-term sickness absence, with similar findings in both countries (table 2). The risk of disability pension, on the other hand, showed some variations by host country of resettlement. Among refugees with 2–5 years of residence, disability pension risk was higher in Sweden (HR_adj_ 1.65, 95% CI 2.34–2.04) and lower in Norway (HR_adj_ 0.79, 95% CI 0.67–0.94), compared to the host populations.

Although belonging to the group with fewer years of length of stay in Norway or Sweden had generally showed a higher risk of long-term unemployment, the stratum-specific risk estimates showed much variations for specific birth country groups across both host countries in the analysis for interaction between refugees’ birth country and length of stay (supplementary table S4). The results for long-term sickness absence or disability pension were statistically non-significant for most birth country and length of stay groups. The proportion of refugees by birth country and length of stay for both host countries is shown in supplementary figure S3.

## Discussion

### Main findings

The incidence rates of long-term unemployment, long-term sickness absence and disability pension were generally higher among refugee groups compared to the corresponding host populations, except for disability pension among refugees from African countries. Refugees in Norway generally had lower incidence rates of long-term unemployment and higher incidence rates of long-term sickness absence and disability pension than refugees in Sweden. However, compared to the host populations, refugees in Norway had a comparatively higher relative risk (HR_adj_) of long-term unemployment and lower relative risk of disability pension than refugees in Sweden. Although the relative risks of the different labor market marginalization measures varied by refugees’ birth country, these generally were similar for a specific birth country group resettling in Sweden versus Norway. Regardless of host country of resettlement, refugees from different birth countries generally had similar relative risk of different labor market marginalization measures. Length of stay played a strong modifying role regarding the risk of long-term unemployment and disability pension. The shorter the duration, the higher the long-term unemployment risk; this effect was stronger for refugee groups in Sweden than Norway. Meanwhile, a shorter length of stay was associated with a lower risk of disability pension among refugees.

### Long-term unemployment among refugees

In both Sweden and Norway, refugees aged 19–60 years had around four times higher incidence and relative risk of long-term unemployment compared to the respective host population. These results replicate the findings from previous studies on rates of unemployment among adult (13, 39) and young refugees (28) in Sweden (13, 28, 39) and Australia (40). A 2014 study in 25 European Union countries found that unemployment rates among adult refugees were two times higher than their host country peers (41). Several factors, such as job mismatch due to challenges in validating the level of education obtained in the origin country, inadequate language skills, discrimination, and low socioeconomic status, were identified as contributing to higher unemployment among refugees (13, 16, 26).

However, one key difference between our study population and those of previous studies is that we included only refugees with gainful employment at baseline. It is, therefore, a novel finding that even among refugees who had some degree of labor market participation at baseline, long-term unemployment risk in the subsequent years is much higher compared with their host population. These results may suggest that refugees with some labor market attachment in Sweden and Norway are disproportionately more vulnerable to adverse labor market outcomes than the respective host populations. Thus, specific policies are warranted to improve the labor market attachment among refugees with gainful employment in both these host countries.

In accordance with findings from previous studies (13, 39), we found a much higher risk of long-term unemployment among refugees from Somalia, Afghanistan, Iraq and Syria than the other birth country groups. This study further indicates that, compared to the host populations, refugees from Afghanistan and Iraq seem to have been relatively less marginalized regarding long-term unemployment in the Norwegian labor market than Sweden, though they are still at a higher risk than the host population. Future research should identify what factors contributed to a lower relative risk of long-term unemployment in Norway than Sweden and how these factors can be utilized for the labor market integration of specific refugee groups in these host countries.

### Long-term sickness absence among refugees

With the exception of refugees from Somalia who resettled in Sweden, all other refugee groups in both host countries had higher incidence rates of long-term sickness absence than the host populations.

It was previously reported that Somalian refugees, when sick, often consult their community contacts, ie, family, acquaintances etc, before seeking help from formal healthcare services (18). As longer-term sick leave requires certification from a physician, this differential healthcare-seeking practices of refugees from Somalia may have influenced their rates of long-term sickness absence. It is also possible that those who manage to get and keep a job among refugees from Somalia tend to be healthier and therefore, have lower long-term sickness absence. Among the initial cohort of refugees from Somalia, 71% and 62% were excluded for not having gainful employment at baseline in Sweden and Norway, respectively (data not shown), which was much higher than the same proportion among all refugees (46% and 29%). Refugees from Somalia also had a high incidence of long-term unemployment, which suggests that fewer individuals remained employed during the follow-up period, thus reducing long-term sickness absence risk. The relative risk of long-term sickness absence was generally higher among the refugee groups in both Sweden and Norway. However, this was not the case for refugees from African countries. These discrepancies in the results by birth country may arise from the level of education and health literacy as well as labor market attachment in the host country. Moreover, knowledge of the healthcare and social insurance regulations also influences the process of certification for sickness absence. Refugees from African countries were reported to be vulnerable and less knowledgeable in these regards (13, 42), which may have contributed to these findings.

### Disability pension among refugees

Similar to the findings regarding long-term sickness absence, incidence of disability pension was generally higher among refugee groups in both Sweden and Norway than their host country peers. Exceptions were refugees from African countries who had a lower incidence of disability pension in both Sweden and Norway. Similar factors as for the risk of long-term sickness absence could contribute to these results. As the receipt of disability pension happens after a long medical evaluation process, a lower educational level and inadequate knowledge of the host country’s healthcare system and social insurance regulations among refugees from Africa (13, 42) may put them in such disadvantageous circumstances.

### Labor market marginalization among refugees and host country of resettlement

Both the host population and refugee groups in Norway had lower incidence of long-term unemployment and higher incidence rates of long-term sickness absence and disability pension than their counterparts in Sweden. These results were expected due to the much lower unemployment rates (23) and more generous social insurance regulations in Norway than Sweden. When comparing the relative risk, refugee groups generally had a similar risk of the three labor market marginalization measures in the two host countries. These findings may suggest that non-structural factors that are related to the refugee experience such as socioeconomic status and health conditions, rather than structural factors in the host country, could be more important in explaining the relative risk of labor market marginalization among refugees compared to Swedish-born and Norwegian-born host populations.

### Labor market marginalization among refugees and length of stay

In both Sweden and Norway, a longer length of stay was associated with a lower risk of long-term unemployment and a higher risk of disability pension. These results are in line with previous studies on the risk of labor market marginalization among refugees in Sweden (28, 39). Refugees with a longer length of stay have more opportunities to develop their language skills and get acquainted with the labor market, which improves their chances of labor market participation. A longer length of stay also helps in gaining access to and understanding of the healthcare and social insurance systems of the host country, which influences the receipt of labor market marginalization measures.

The influence of length of stay on long-term unemployment risk was much stronger among refugees in Sweden than Norway. This novel finding in our study showed that the difference between long-term unemployment risk among those with the shortest versus the longest length of stay was much more prominent in Sweden than Norway. In both countries, a shorter length of stay was associated with a higher risk of long-term unemployment and a lower risk of disability pension compared to a longer length of stay. However, the shortest length of stay was associated with a higher relative risk of disability pension for refugees in Sweden, but a lower relative risk for refugees in Norway. Moreover, we found a positive interaction between refugees’ birth country and length of stay regarding long-term unemployment risk and a negative interaction regarding disability pension risk. The gradient of this effect for long-term unemployment was more prominent among the same birth country group who resettled in Sweden than in Norway. A report on immigrants’ integration into the Nordic labor market highlighted that Norway’s integration measures for newly-arrived refugees are more efficient than Sweden's (25). However, this initial advantage among refugees in Norway compared to Sweden becomes less apparent when comparing the long-term labor market outcomes for refugees in these host countries (25). Our comparative analyses between Sweden and Norway are in line with these findings. It is, therefore, important to review the integration policies for newly-arrived refugees, particularly in Sweden, to improve the labor market attachment among them.

### Strengths and limitations

A longitudinal study design using high-quality registers covering the entire population in Sweden and Norway is the main strength of this study which limits bias from non-response or selective loss to follow-up. To the authors’ knowledge, this is also the first study comparing risk of labor market marginalization among refugees from the same origin country who resettled in different host countries. However, our results should be interpreted in light of the study’s limitations. Firstly, differences in the social insurance regulations in Sweden and Norway may lead to differential measurement of the labor market marginalization outcomes by host country. A long history of collaborative research between Sweden and Norway helps to minimize bias from this source by harmonizing the data in the most efficient way. While inclusion of only gainfully employed individuals during November 2009 facilitated comparisons between refugees and the host population, it may have introduced selection bias. Refugees who manage to find employment can be healthier and have a higher educational level. Consequently, our results are not generalizable to all refugees in Sweden and Norway. Additionally, our results are primarily generalizable among resettled refugees in high-income countries and not to asylum seekers with limited-to-no access to social insurance benefits or refugees resettled in countries with social insurance regulations significantly different to those of Sweden and Norway.

### Concluding remarks

The novel findings in this study showed that refugee groups from the same birth country resettling in two different host countries (Norway and Sweden) generally had very comparable relative risk estimates of labor market marginalization measures. These findings hint towards the importance of factors related to the refugee experience, such as socioeconomic status and health conditions, rather than structural factors in the host country for the risk of labor market marginalization among refugees. Shorter length of stay had a strong negative influence on the risk of labor market marginalization among refugees in Sweden and Norway, which warrants more efficient labor market integration policies for newly-arrived refugees.

## Supplementary material

Supplementary material

## Data Availability

The data used in this study cannot be made publicly available due to privacy regulations. According to the General Data Protection Regulation, the Swedish law SFS 2018:218, the Swedish Data Protection Act, the Swedish Ethical Review Act, and the Public Access to Information and Secrecy Act, these types of sensitive data can only be made available for specific purposes, including research, that meets the criteria for access to this sort of sensitive and confidential data as determined by a legal review. Readers may contact Professor Kristina Alexanderson (kristina.alexanderson@ki.se) regarding the data in Sweden and Professor Ingrid Sivesind Mehlum (ingrid.s.mehlum@stami.no) regarding the data in Norway.
