# Supplementary material for: Does country of resettlement influence the risk of labor market marginalization among refugees? A cohort study in Sweden and Norway
Source: Scand J Work Environ Health. 2024 Apr 29;50(4):279–89. doi: 10.5271/sjweh.4154 (PMC11129853; doi:10.5271/sjweh.4154)

Does country of resettlement influence the risk of labor market marginalization among refugees? A cohort study in Sweden and Norway[1]

by Ridwanul Amin, PhD,[2] Ellenor Mittendorfer-Rutz, PhD, Karina Undem, MPh, Ingrid Sivesind Mehlum, PhD, Rachel Louise Hasting, PhD

1. *Supplementary material*
2. *Correspondence to: Ridwanul Amin, Karolinska Institutet, Department of Clinical Neuroscience, Division of Insurance Medicine, Stockholm, Sweden. [E-mail: ridwanul.amin@ki.se]*

Contents

The social insurance systems in Sweden and Norway 2

Supplementary Figure S1. Flow-chart of the selection of the study population in Sweden and Norway. 4

Supplementary Figure S2. Directed acyclic graph of the associations among the exposure, outcome and the covariates. 5

Supplementary Figure S3. Proportion (%) of individuals among gainfully employed refugees by birth country and length of stay (years) across host countries (Sweden and Norway). 6

Supplementary Table S1: Risk of long-term unemployment from 2010-2016 among gainfully employed refugees from different regions and countries[a] of birth who resettled in Sweden and Norway, compared with the Swedish-born and Norwegian-born population, respectively; crude and multivariate hazard ratios (HRs) with 95% confidence intervals (CIs). 7

Supplementary Table S2: Risk of long-term sickness absence from 2010-2016 among gainfully employed refugees from different regions and countries[a] of birth who resettled in Sweden and Norway, compared with the Swedish-born and Norwegian-born population, respectively; crude and multivariate hazard ratios (HRs) with 95% confidence intervals (CIs). 9

Supplementary Table S3: Risk of disability pension from 2010-2016 among gainfully employed refugees from different regions and countries[a] of birth who resettled in Sweden and Norway, compared with the Swedish-born and Norwegian-born population, respectively; crude and multivariate hazard ratios (HRs) with 95% confidence intervals (CIs). 11

Supplementary Table S4: Test of interaction between birth country and length of stay in the risk of long-term unemployment, long-term sickness absence and disability pension during 2010-2016 among gainfully employed refugees from different birth countries[a] who resettled in Sweden and Norway. 13

Supplementary Figure S4: Incidence rates of long-term unemployment among gainfully employed refugees by birth country and length of stay (years) across host countries (Sweden and Norway). 15

Supplementary Figure S5: Incidence rates of long-term sickness absence among gainfully employed refugees by birth country and length of stay (years) across host countries (Sweden and Norway). 16

Supplementary Figure S6: Incidence rates of disability pension among gainfully employed refugees by birth country and length of stay (years) across host countries (Sweden and Norway). 17

The social insurance systems in Sweden and Norway

Both the Swedish and Norwegian social insurance systems are public and aim at providing financial security to the country's residents. In Sweden, unemployment insurance comprises basic insurance and income-related optional insurance. All individuals aged 20-64 years in Sweden who are not covered by the voluntary income-related insurance are entitled to basic unemployment benefit provided that certain preconditions are met, i.e. they are registered at the National Public Employment Service to seek jobs and ready to enter the labor market if suitable jobs are offered (1). Unemployment benefits in Sweden cover 80% of an individual's lost income; with an upper limit of 910 SEK per day for the first 200 days of unemployment and then, 70% until day 300 (1).

From the age of 16, all individuals in Sweden are allowed sickness absence benefits if their work capacity is reduced at least 25% due to disease or injury (27). These benefits amount up to 80% of lost income from work or unemployment benefits. The first 14 days, excluding one qualifying day of sickness absence benefit, is paid by the employer. A physician's certificate is required from day 8. Sick leave periods extending more than 14 days are compensated by the Social Insurance Agency (1). sickness absence benefits can be claimed full- or part-time (i.e. 25%, 50% or 75%). All individuals 30-64 years of age with permanent impairment of work capacity due to disease or injury are compensated by a permanent disability pension in Sweden (1). On the other hand, individuals 19-29 years of age can be granted temporary disability pension where work disability is expected to last a minimum of one year or if they cannot complete their compulsory education due to health issues (1, 2).

After fulfilling similar criteria for unemployment insurance as in Sweden, individuals up to age 67 in Norway may get full or partial unemployment benefits for up to 2 years depending on previous income from work. The benefits cover 62.4% of previous income up to 6 times the basic amount (111 477 NOK in 2022) (3, 4). Individuals younger than 70 years who have lost at least 20% of their income due to illness or injury and were in paid employment the 4 weeks beforehand are entitled to sickness absence benefits (5). The employer pays for the first 16 calendar days, thereafter payments are reimbursed by the Norwegian Labor and Welfare Administration (2). Sickness absence benefits equal 100% of the lost income up to 6 times the basic amount, and are paid for a maximum of one year, with a possibility to move to work assessment

allowance for a maximum of 5 years if the individual is still unable to work due to illness (6). Sickness absence benefits can be claimed full- or part-time, from 20-100%. Individuals aged 18-67 years who have a permanent reduction of at least 50% of their work capacity due to disease or injury (30% if this is recognised as occupational) are entitled to disability pension in Norway (2).

References:

1. Ludvigsson JF, Svedberg P, Olén O, Bruze G, Neovius M. The longitudinal integrated database for health insurance and labor market studies (LISA) and its use in medical research. Eur J Epidemiol. 2019;34(4):423-37.
2. Norwegian Ministry of Labor and Social Affairs. The Norwegian Social Insurance Scheme 2022 Norway2022 [18 October 2022]. Available from: https://www.regjeringen.no/en/dokumenter/the-norwegian-social-insurance-scheme-2020/id2478621/.
3. Niederkrotenthaler T, Wang M, Helgesson M, Wilcox H, Gould M, Mittendorfer-Rutz E. Labour market marginalization subsequent to suicide attempt in young migrants and native Swedes. Soc Psychiatry Psychiatr Epidemiol. 2017;52(5):549-58.
4. Norwegian Labor and Welfare Administration. Søk om penger - arbeidsledig (Appy for benefit - Unemployed) 2021 [07 September 2021]. Available from: https://www.nav.no/arbeid/arbeidsledig#kort-fortalt.
5. Norwegian Labor and Welfare Administration. Sykepenger (Sick pay) 2022 [07 December 2022]. Available from: https://www.nav.no/no/person/arbeid/sykmeldt-arbeidsavklaringspenger-og-yrkesskade/sykepenger/sykepenger-til-arbeidstakere.
6. Norwegian Labor and Welfare Administration. Work assessment allowance 2022 [07 December 2022]. Available from: https://www.nav.no/aap/en.

Supplementary Figure S1. Flow-chart of the selection of the study population in Sweden and Norway.

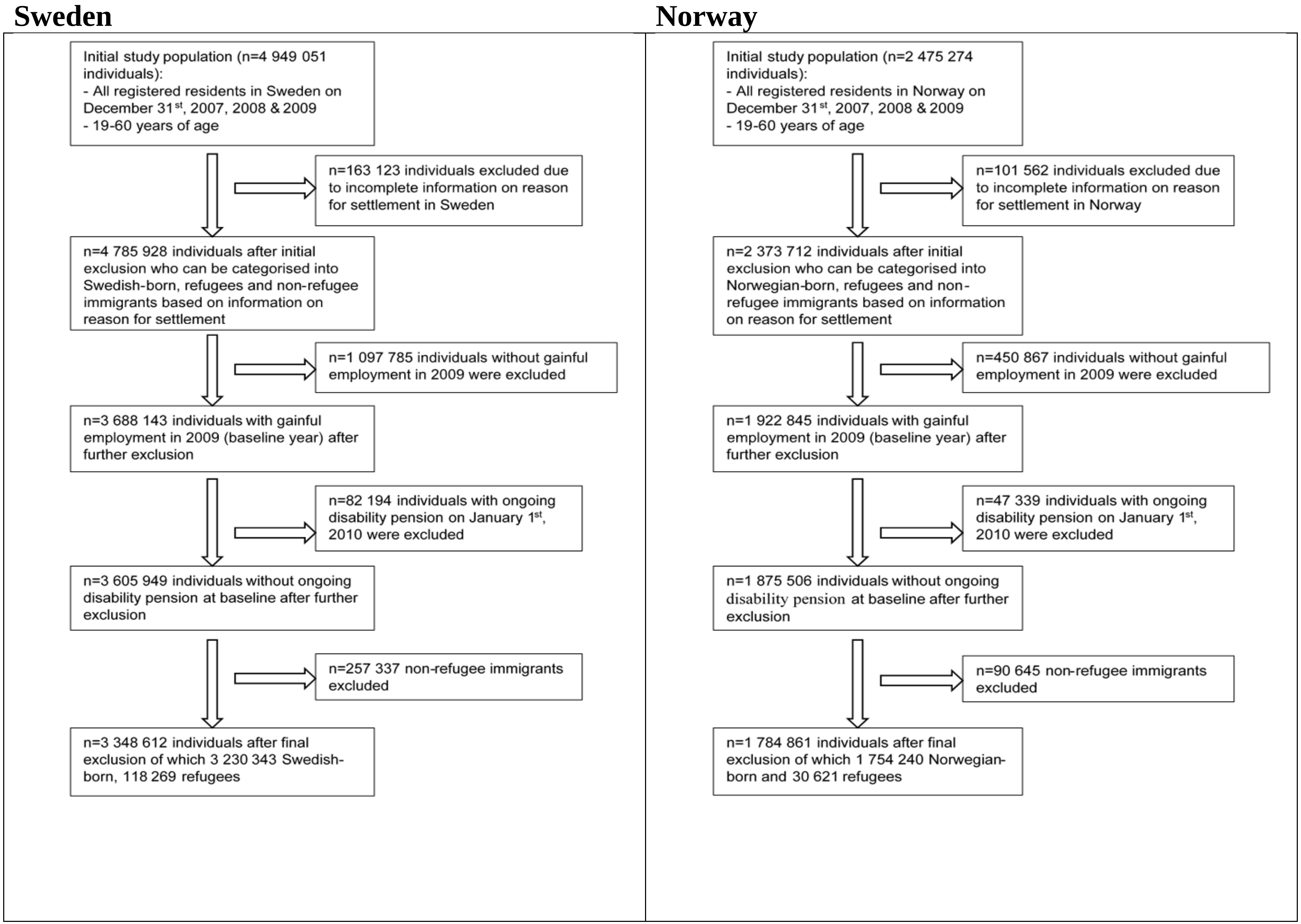

Supplementary Figure S2. Directed acyclic graph of the associations among the exposure, outcome and the covariates.

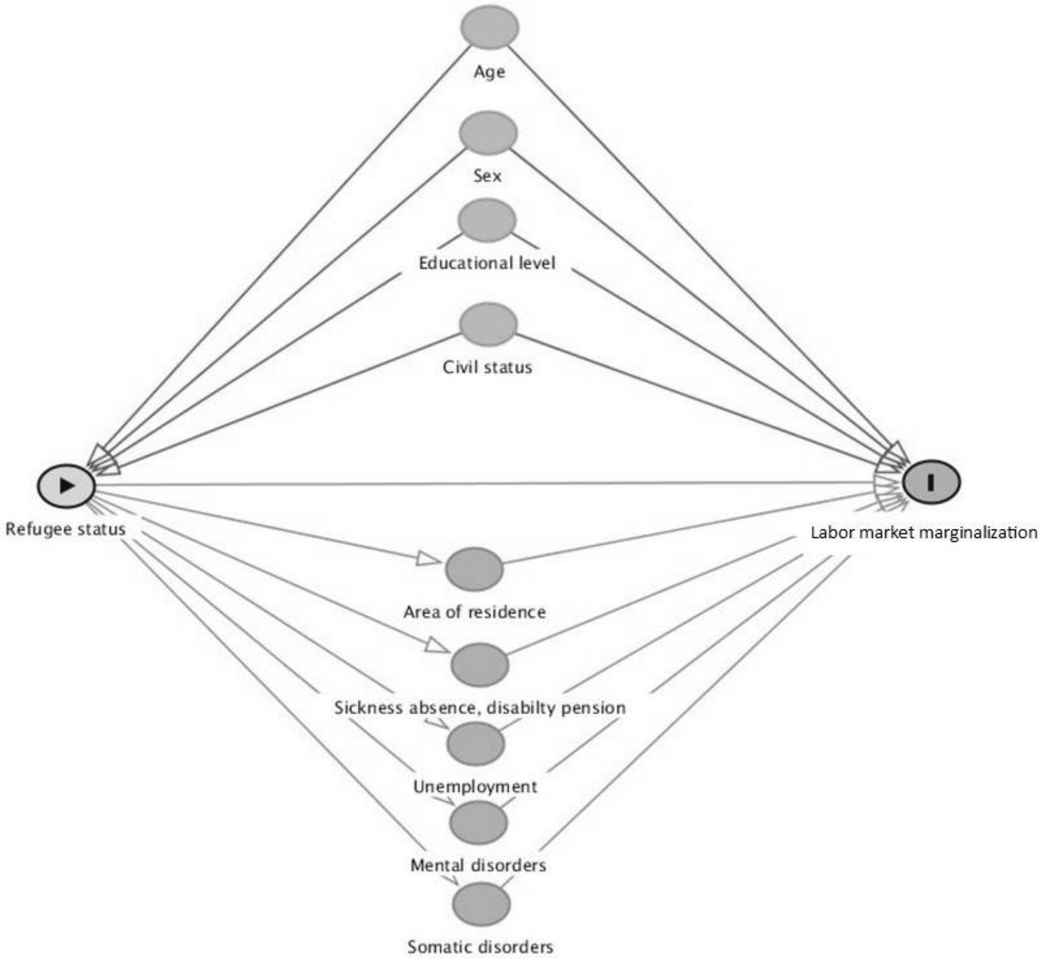

Supplementary Figure S3. Proportion (%) of individuals among gainfully employed refugees by birth country and length of stay (years) across host countries (Sweden and Norway).

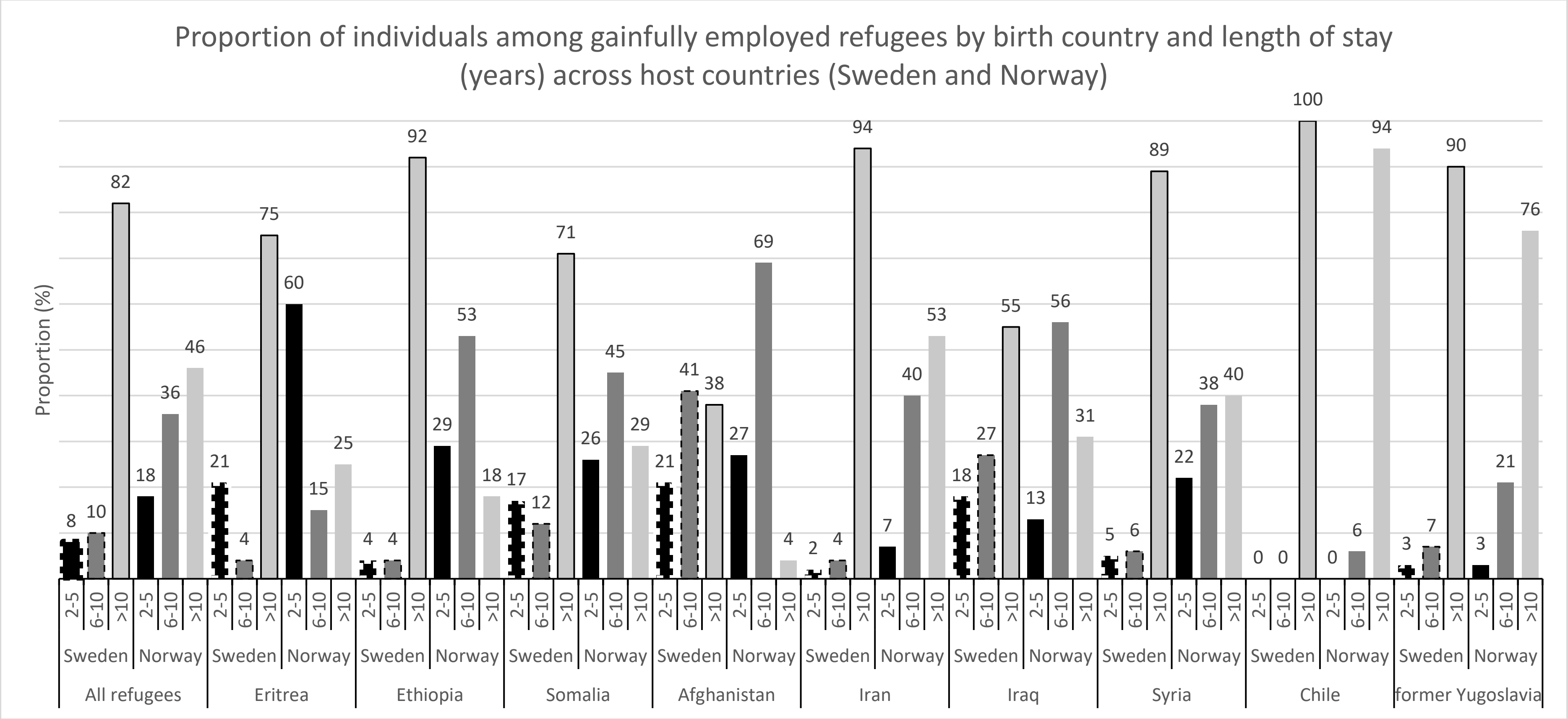

Supplementary Table S1: Risk of long-term unemployment from 2010-2016 among gainfully employed refugees from different regions and countries[a] of birth who resettled in Sweden and Norway, compared with the Swedish-born and Norwegian-born population, respectively; crude and multivariate hazard ratios (HRs) with 95% confidence intervals (CIs).

	Long-term unemployment n events (%)	Long-term unemployment rate/1000 person-years	Crude HR (CI)	Adjusted[b] HR (CI)
**Swedish-born**	133 073 (4.1)	8.1	1	1
**Refugees in Sweden**	15 628 (13.2)	29.0	3.41 (3.36–3.47)	3.44 (3.38–3.50)
Africa (region)	1468 (14.3)	30.2	3.61 (3.43–3.80)	3.43 (3.26–3.61)
Eritrea	240 (11.1)	23.5	2.80 (2.47–3.18)	2.43 (2.14–2.76)
Ethiopia	269 (9.9)	20.5	2.47 (2.19–2.79)	2.34 (2.08–2.64)
Somalia	589 (19.9)	42.9	5.07 (4.67–5.49)	4.85 (4.47–5.26)
Other countries in Africa	370 (15.4)	32.2	3.85 (3.48–4.26)	4.07 (3.67–4.51)
Asia (region)	8334 (17.0)	38.3	4.46 (4.37–4.56)	4.49 (4.39–4.59)
Afghanistan	314 (17.3)	37.1	4.39 (3.93–4.91)	4.67 (4.18–5.21)
Iran	1759 (11.2)	24.7	2.91 (2.78–3.05)	3.04 (2.90–3.18)
Iraq	3830 (22.0)	52.0	5.94 (5.75–6.13)	6.09 (5.89–6.29)
Syria	609 (18.0)	40.6	4.70 (4.34–5.09)	4.53 (4.18–4.90)
Other countries in Asia	1822 (17.0)	36.9	4.35 (4.16–4.56)	4.20 (4.01–4.40)
South America (region)	710 (9.5)	21.5	2.51 (2.33–2.70)	2.23 (2.07–2.40)
Chile	480 (8.9)	20.4	2.37 (2.17–2.59)	2.02 (1.85–2.21)
Other countries in South America	230 (11.1)	24.0	2.84 (2.50–3.24)	2.86 (2.52–3.26)
Other regions	5116 (9.9)	21.3	2.53 (2.46–2.60)	2.67 (2.60–2.75)
Former Yugoslavia	4004 (9.6)	20.6	2.44 (2.37–2.52)	2.61 (2.53–2.70)
Other countries	1112 (11.3)	24.5	2.91 (2.74–3.08)	2.99 (2.82–3.17)
**Norwegian-born**	52 807 (3.0)	5.4	1	1
**Refugees in Norway**	3977 (13.0)	26.2	4.67 (4.52–4.83)	3.70 (3.58–3.82)
Africa (region)	1173 (18.5)	36.3	6.52 (6.15–6.91)	4.42 (4.17–4.69)
Eritrea	94 (14.8)	27.4	4.93 (4.02–6.03)	3.00 (2.45–3.68)
Ethiopia	76 (9.4)	17.3	3.13 (2.50–3.93)	2.27 (1.81–2.84)
Somalia	640 (23.1)	48.5	8.67 (8.02–9.37)	5.17 (4.77–5.60)
Other countries in Africa	363 (17.2)	32.1	5.78 (5.22–6.41)	4.82 (4.34–5.34)
Asia (region)	1744 (13.9)	28.1	5.00 (4.77–5.24)	3.68 (3.51–3.87)
Afghanistan	352 (17.4)	34.7	6.19 (5.57–6.87)	3.76 (3.38–4.19)

	Long-term unemployment n events (%)	Long-term unemployment rate/1000 person-years	Crude HR (CI)	Adjusted[b] HR (CI)
Iran	261 (11.1)	23.1	4.08 (3.61–4.60)	3.41 (3.02–3.85)
Iraq	590 (15.7)	34.3	6.03 (5.56–6.53)	4.60 (4.24–4.99)
Syria	16 (11.4)	24.9	4.36 (2.67–7.11)	3.98 (2.44–6.50)
Other countries in Asia	525 (12.2)	23.1	4.16 (3.81–4.53)	3.10 (2.85–3.38)
South America (region)	23 (10.2)	20.4	3.65 (2.42–5.49)	3.08 (2.04–4.63)
Chile	7 (13.0)	31.9	5.38 (2.56–11.28)	3.55 (1.69–7.44)
Other countries in South America	16 (9.3)	17.7	3.20 (1.96–5.22)	2.91 (1.79–4.76)
Other regions	1037 (9.0)	18.4	3.28 (3.08–3.49)	3.18 (2.99–3.38)
Former Yugoslavia	739 (7.5)	15.2	2.71 (2.52–2.92)	2.69 (2.50–2.90)
Other countries	298 (17.9)	38.8	6.79 (6.06–7.61)	5.81 (5.18–6.51)

[a] Countries which generated the largest number of refugees to Sweden and Norway.
[b] Adjusted for sex, age, educational level and civil status.

Supplementary Table S2: Risk of long-term sickness absence from 2010-2016 among gainfully employed refugees from different regions and countries[a] of birth who resettled in Sweden and Norway, compared with the Swedish-born and Norwegian-born population, respectively; crude and multivariate hazard ratios (HRs) with 95% confidence intervals (CIs).

	Long-term sickness absence n events (%)	Long-term sickness absence rate/1000 person-years	Crude HR (CI)	Adjusted[b] HR (CI)
**Swedish-born**	367 216 (11.6)	19.0	1	1
**Refugees in Sweden**	18 185 (15.9)	25.8	1.35 (1.33–1.37)	1.40 (1.38–1.42)
Africa (region)	1178 (11.7)	18.5	0.97 (0.91–1.02)	0.96 (0.91–1.02)
Eritrea	302 (14.3)	22.8	1.20 (1.07–1.34)	1.06 (0.95–1.19)
Ethiopia	328 (12.3)	19.4	1.02 (0.91–1.13)	0.98 (0.88–1.09)
Somalia	286 (9.8)	15.3	0.80 (0.71–0.90)	0.82 (0.73–0.92)
Other countries in Africa	262 (11.1)	17.6	0.92 (0.82–1.04)	1.01 (0.90–1.15)
Asia (region)	7281 (15.3)	24.8	1.30 (1.27–1.33)	1.44 (1.41–1.48)
Afghanistan	219 (12.3)	19.5	1.02 (0.90–1.17)	1.31 (1.15–1.49)
Iran	2555 (16.9)	28.0	1.47 (1.42–1.53)	1.60 (1.54–1.67)
Iraq	2462 (14.5)	23.4	1.23 (1.18–1.28)	1.46 (1.41–1.52)
Syria	560 (17.2)	27.7	1.45 (1.34–1.58)	1.46 (1.34–1.58)
Other countries in Asia	1485 (14.2)	22.7	1.19 (1.13–1.25)	1.22 (1.16–1.28)
South America (region)	1139 (15.7)	26.4	1.39 (1.31–1.48)	1.25 (1.18–1.32)
Chile	873 (16.7)	28.3	1.49 (1.40–1.60)	1.29 (1.20–1.37)
Other countries in South America	266 (13.2)	21.6	1.13 (1.01–1.28)	1.13 (1.00–1.27)
Other regions	8587 (17.2)	28.1	1.48 (1.45–1.51)	1.47 (1.44–1.51)
Former Yugoslavia	7216 (17.9)	29.1	1.53 (1.49–1.56)	1.53 (1.49–1.56)
Other countries	1371 (14.4)	23.8	1.25 (1.19–1.32)	1.25 (1.18–1.32)
**Norwegian-born**	357 008 (21.5)	37.7	1	1
**Refugees in Norway**	8711 (30.8)	55.4	1.48 (1.45–1.51)	1.44 (1.41–1.47)
Africa (region)	1432 (23.9)	40.0	1.08 (1.02–1.14)	0.96 (0.92–1.02)
Eritrea	136 (22.6)	37.2	0.99 (0.84–1.18)	0.77 (0.65–0.91)
Ethiopia	208 (27.1)	46.2	1.23 (1.08–1.41)	1.10 (0.96–1.26)
Somalia	663 (25.7)	43.7	1.19 (1.10–1.28)	1.03 (0.95–1.11)
Other countries in Africa	425 (20.8)	34.1	0.92 (0.83–1.01)	0.89 (0.81–0.98)
Asia (region)	3672 (31.8)	57.2	1.53 (1.48–1.58)	1.53 (1.48–1.58)
Afghanistan	549 (29.0)	49.7	1.33 (1.22–1.45)	1.40 (1.29–1.53)
Iran	777 (36.7)	69.6	1.84 (1.72–1.98)	1.88 (1.75–2.01)

	Long-term sickness absence n events (%)	Long-term sickness absence rate/1000 person-years	Crude HR (CI)	Adjusted[b] HR (CI)
Iraq	1223 (36.4)	69.2	1.84 (1.74–1.95)	2.04 (1.93–2.16)
Syria	50 (40.7)	81.0	2.14 (1.62–2.83)	2.39 (1.81–3.15)
Other countries in Asia	1073 (26.4)	45.4	1.21 (1.14–1.29)	1.11 (1.04–1.17)
South America (region)	70 (32.6)	60.6	1.61 (1.27–2.03)	1.45 (1.14–1.83)
Chile	23 (45.1)	95.5	2.50 (1.66–3.77)	2.16 (1.44–3.25)
Other countries in South America	47 (28.7)	51.4	1.37 (1.03–1.82)	1.25 (0.94–1.66)
Other regions	3537 (33.7)	63.0	1.67 (1.61–1.72)	1.65 (1.60–1.71)
Former Yugoslavia	3021 (33.6)	63.0	1.67 (1.61–1.73)	1.67 (1.62–1.74)
Other countries	516 (33.9)	62.6	1.66 (1.53–1.81)	1.53 (1.40–1.67)

[a] Countries which generated the largest number of refugees to Sweden and Norway.
[b] Adjusted for sex, age, educational level and civil status.

Supplementary Table S3: Risk of disability pension from 2010-2016 among gainfully employed refugees from different regions and countries[a] of birth who resettled in Sweden and Norway, compared with the Swedish-born and Norwegian-born population, respectively; crude and multivariate hazard ratios (HRs) with 95% confidence intervals (CIs).

	Disability pension n events (%)	Disability pension rate/1000 person-years	Crude HR (CI)	Adjusted[b] HR (CI)
**Swedish-born**	28 021 (0.9)	1.4	1	1
**Refugees in Sweden**	2449 (2.1)	3.1	2.31 (2.22–2.41)	2.45 (2.35–2.56)
Africa (region)	79 (0.8)	1.2	0.85 (0.68–1.06)	0.88 (0.71–1.10)
Eritrea	16 (0.7)	1.1	0.82 (0.50–1.33)	0.69 (0.42–1.12)
Ethiopia	18 (0.7)	0.9	0.72 (0.46–1.15)	0.68 (0.43–1.07)
Somalia	24 (0.8)	1.2	0.89 (0.60–1.33)	1.10 (0.74–1.65)
Other countries in Africa	21 (0.9)	1.3	0.96 (0.63–1.48)	1.18 (0.77–1.81)
Asia (region)	790 (1.6)	2.4	1.79 (1.67–1.92)	2.03 (1.89–2.17)
Afghanistan	17 (0.9)	1.4	1.03 (0.64–1.65)	1.59 (0.99–2.56)
Iran	305 (1.9)	3.0	2.20 (1.97–2.46)	2.24 (2.00–2.51)
Iraq	215 (1.2)	1.9	1.37 (1.20–1.57)	1.80 (1.57–2.06)
Syria	62 (1.8)	2.7	2.01 (1.57–2.58)	2.07 (1.61–2.66)
Other countries in Asia	191 (1.8)	2.7	1.96 (1.70–2.25)	2.05 (1.77–2.36)
South America (region)	94 (1.3)	2.0	1.45 (1.18–1.78)	1.13 (0.92–1.39)
Chile	73 (1.4)	2.1	1.56 (1.24–1.97)	1.13 (0.90–1.42)
Other countries in South America	21 (1.0)	1.6	1.16 (0.75–1.78)	1.13 (0.73–1.73)
Other regions	1486 (2.9)	4.3	3.21 (3.05–3.38)	3.41 (3.24–3.60)
Former Yugoslavia	1347 (3.2)	4.8	3.57 (3.38–3.77)	3.90 (3.69–4.12)
Other countries	139 (1.4)	2.2	1.62 (1.37–1.92)	1.56 (1.32–1.84)
**Norwegian-born**	64 095 (3.7)	5.7	1	1
**Refugees in Norway**	1550 (5.1)	7.5	1.32 (1.26–1.39)	1.57 (1.49–1.66)
Africa (region)	146 (2.3)	3.3	0.59 (0.50–0.69)	0.73 (0.62–0.86)
Eritrea	18 (2.8)	4.1	0.72 (0.45–1.14)	0.66 (0.41–1.04)
Ethiopia	22 (2.7)	3.9	0.69 (0.45–1.04)	0.91 (0.60–1.38)
Somalia	58 (2.1)	3.0	0.54 (0.42–0.69)	0.63 (0.49–0.82)
Other countries in Africa	48 (2.3)	3.3	0.58 (0.44–0.77)	0.82 (0.62–1.10)
Asia (region)	655 (5.2)	7.7	1.35 (1.25–1.46)	1.65 (1.53–1.79)
Afghanistan	44 (2.2)	3.1	0.55 (0.41–0.74)	0.91 (0.68–1.23)
Iran	183 (7.8)	11.5	2.03 (1.75–2.34)	2.37 (2.05–2.74)
Iraq	221 (5.9)	8.7	1.53 (1.34–1.75)	2.29 (2.00–2.61)

	Disability pension n events (%)	Disability pension rate/1000 person-years	Crude HR (CI)	Adjusted[b] HR (CI)
Syria	14 (9.9)	14.9	2.62 (1.55–4.43)	3.21 (1.90–5.42)
Other countries in Asia	193 (4.5)	6.6	1.16 (1.01–1.34)	1.13 (0.98–1.30)
South America (region)	10 (4.4)	6.6	1.16 (0.63–2.16)	1.00 (0.54–1.86)
Chile	2 (3.7)	5.5	0.96 (0.24–3.84)	0.88 (0.22–3.50)
Other countries in South America	8 (4.7)	6.9	1.23 (0.61–2.45)	1.04 (0.52–2.07)
Other regions	739 (6.4)	9.7	1.71 (1.59–1.84)	1.93 (1.79–2.07)
Former Yugoslavia	657 (6.7)	10.1	1.79 (1.65–1.93)	2.02 (1.87–2.18)
Other countries	82 (4.9)	7.3	1.29 (1.04–1.60)	1.38 (1.11–1.72)

[a] Countries which generated the largest number of refugees to Sweden and Norway.
[b] Adjusted for sex, age, educational level and civil status.

Supplementary Table S4: Test of interaction between birth country and length of stay in the risk of long-term unemployment, long-term sickness absence and disability pension during 2010-2016 among gainfully employed refugees from different birth countries[a] who resettled in Sweden and Norway.

	**Long–term unemployment**			**Long–term sickness absence**			**Disability pension**		
**Birth country**	Length of stay 2–5 years vs 6–10 years HR (CI)[b]	Length of stay 2–5 years vs >10 years HR (CI)[b]	Length of stay 6–10 years vs >10 years HR (CI)[b]	Length of stay 2–5 years vs 6–10 years HR (CI)[b]	Length of stay 2–5 years vs >10 years HR (CI)[b]	Length of stay 6–10 years vs >10 years HR (CI)[b]	Length of stay 2–5 years vs 6–10 years HR (CI)[b]	Length of stay 2–5 years vs >10 years HR (CI)[b]	Length of stay 6–10 years vs >10 years HR (CI)[b]
**Refugees in Sweden**									
Eritrea	**2.85 (1.27–6.41)**	**2.45 (1.80–3.34)**	1.06 (0.49–2.28)	0.83 (0.42–1.65)	0.86 (0.62–1.21)	1.00 (0.55–1.80)	–[c]	1.32 (0.16–10.6)	–[c]
Ethiopia	**2.60 (1.21–5.58)**	**2.68 (1.72–4.17)**	1.20 (0.65–2.21)	0.45 (0.14–1.39)	0.40 (0.16–1.00)	0.86 (0.47–1.59)	–[c]	–[c]	2.95 (0.37–23.3)
Somalia	**1.67 (1.28–2.17)**	**2.44 (1.97–3.01)**	**1.51 (1.17–1.95)**	0.77 (0.48–1.26)	0.83 (0.56–1.22)	0.98 (0.68–1.42)	–[c]	1.29 (0.35–4.73)	–[b]
Afghanistan	**1.45 (1.08–1.94)**	**2.32 (1.64–3.28)**	**1.62 (1.21–2.16)**	0.71 (0.45–1.11)	0.67 (0.42–1.06)	0.99 (0.74–1.32)	0.17 (0.01–3.21)	0.18 (0.01–3.19)	0.78 (0.26–2.33)
Iran	**1.58 (1.19–2.11)**	**2.25 (1.81–2.81)**	**1.45 (1.19–1.77)**	1.02 (0.76–1.38)	1.14 (0.89–1.46)	1.08 (0.91–1.29)	0.83 (0.30–2.29)	1.20 (0.49–2.92)	1.38 (0.81–2.35)
Iraq	**1.69 (1.55–1.84)**	**2.24 (2.06–2.44)**	**1.33 (1.22–1.44)**	**0.78 (0.68–0.89)**	**0.71 (0.63–0.81)**	0.92 (0.84–1.03)	1.09 (0.64–1.87)	0.96 (0.59–1.56)	0.95 (0.68–1.33)
Syria	1.21 (0.81–1.81)	**1.81 (1.34–2.45)**	**1.53 (1.15–2.03)**	1.33 (0.72–2.45)	0.78 (0.50–1.21)	0.67 (0.45–1.00)	0.81 (0.06–10.6)	0.45 (0.06–2.65)	0.64 (0.16–2.65)
Chile	–[c]	1.04 (0.15–7.40)	1.46 (0.21–10.41)	1.00 (0.02–50.4)	1.15 (0.29–4.62)	2.05 (0.66–6.37)	–[c]	–[c]	–[c]
Former Yugoslavia	**1.36 (1.15–1.61)**	**1.99 (1.72–2.30)**	**1.46 (1.32–1.61)**	0.98 (0.84–1.14)	**1.16 (1.02–1.33)**	**1.18 (1.10–1.28)**	0.81 (0.55–1.19)	1.30 (0.91–1.85)	**1.52 (1.27–1.82)**
**Refugees in Norway**									
Eritrea	1.07 (0.69–1.64)	**1.53 (1.16–2.02)**	1.44 (0.92–2.25)	**0.63 (0.42–0.93)**	**0.67 (0.52–0.86)**	1.07 (0.73–1.56)	0.49 (0.13–1.89)	0.46 (0.20–1.06)	0.93 (0.28–3.10)
Ethiopia	1.11 (0.80–1.54)	**1.57 (1.08–2.30)**	1.41 (1.00–2.00)	1.13 (0.85–1.49)	0.95 (0.72–1.26)	0.85 (0.66–1.09)	0.59 (0.19–1.80)	0.78 (0.25–2.41)	1.33 (0.61–2.91)
Somalia	1.03 (0.90–1.19)	**1.58 (1.35–1.86)**	**1.53 (1.32–1.78)**	**0.75 (0.64–0.89)**	1.02 (0.85–1.22)	**1.35 (1.16–1.57)**	0.65 (0.32–1.33)	0.69 (0.34–1.42)	1.06 (0.64–1.76)
Afghanistan	0.92 (0.77–1.10)	1.14 (0.79–1.66)	1.24 (0.87–1.77)	0.87 (0.73–1.03)	0.93 (0.68–1.28)	1.07 (0.80–1.44)	0.47 (0.20–1.12)	**0.26 (0.09–0.71)**	0.55 (0.27–1.11)
Iran	0.93 (0.68–1.27)	**1.45 (1.07–1.95)**	**1.55 (1.34–1.79)**	1.12 (0.86–1.46)	1.20 (0.93–1.53)	1.07 (0.95–1.20)	1.05 (0.55–1.97)	1.14 (0.62–2.09)	1.09 (0.84–1.42)
Iraq	1.17 (0.99–1.38)	**1.39 (1.16–1.66)**	**1.19 (1.04–1.36)**	0.86 (0.73–1.00)	**1.22 (1.03–1.44)**	**1.42 (1.27–1.59)**	0.61 (0.37–1.00)	**0.51 (0.31–0.84)**	0.84 (0.64–1.08)
Syria	0.56 (0.25–1.23)	1.54 (0.69–3.42)	**2.76 (1.45–5.26)**	1.11 (0.57–2.17)	1.80 (0.96–3.36)	1.62 (0.95–2.78)	0.47 (0.09–2.31)	0.86 (0.17–4.26)	1.84 (0.59–5.71)
Chile	–[c]	–[c]	1.62 (0.97–2.72)	0.82 (0.24–2.73)	0.79 (0.25–2.44)	0.96 (0.63–1.47)	–[c]	–[c]	1.33 (0.49–3.59)
Former Yugoslavia	1.00 (0.76–1.31)	1.20 (0.92–1.57)	**1.21 (1.08–1.34)**	1.02 (0.84–1.24)	1.08 (0.89–1.30)	1.05 (0.98–1.14)	0.48 (0.24–0.98)	**0.49 (0.25–0.99)**	1.02 (0.85–1.22)

[a] Countries which generated the largest number of refugees to Sweden and Norway.
[b]Adjusted for age, sex, educational level and civil status.
[c]Too few (<10) outcome events to estimate HR (CI); The exact number of events is not disclosed due to ethical reasons. HR–Hazard Ratio, CI–Confidence Interval.

Supplementary Figure S4: Incidence rates of long-term unemployment among gainfully employed refugees by birth country and length of stay (years) across host countries (Sweden and Norway).

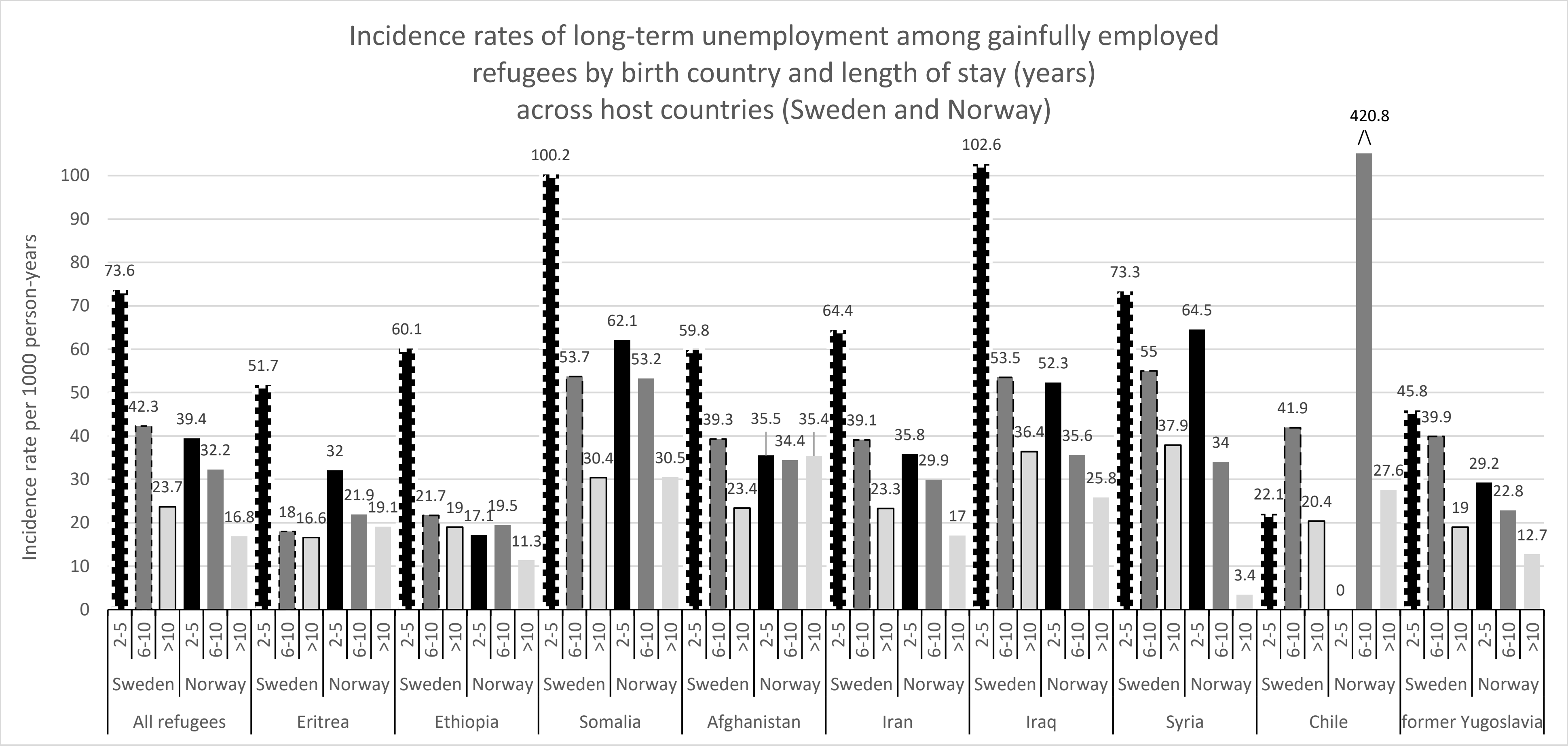

Supplementary Figure S5: Incidence rates of long-term sickness absence among gainfully employed refugees by birth country and length of stay (years) across host countries (Sweden and Norway).

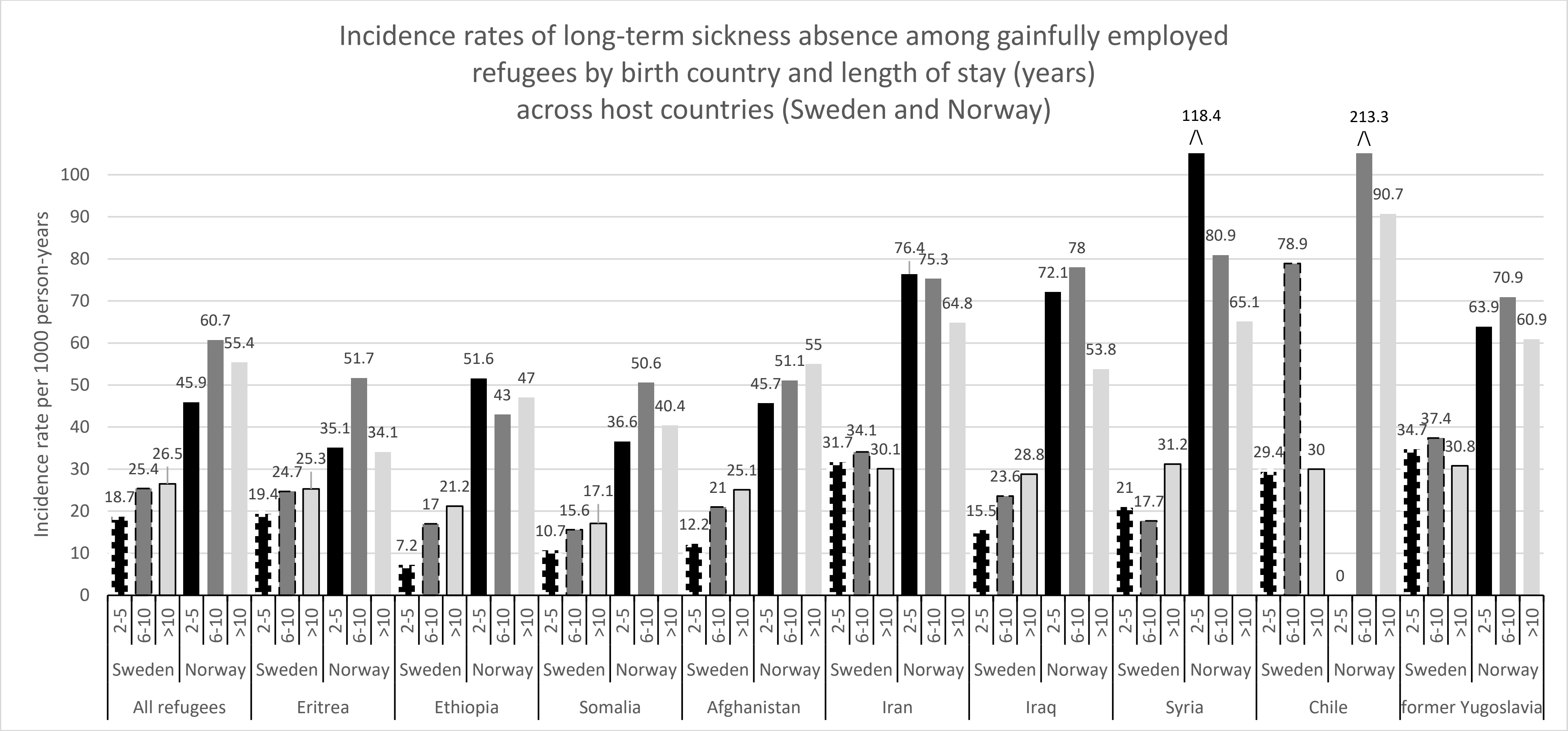

Supplementary Figure S6: Incidence rates of disability pension among gainfully employed refugees by birth country and length of stay (years) across host countries (Sweden and Norway).

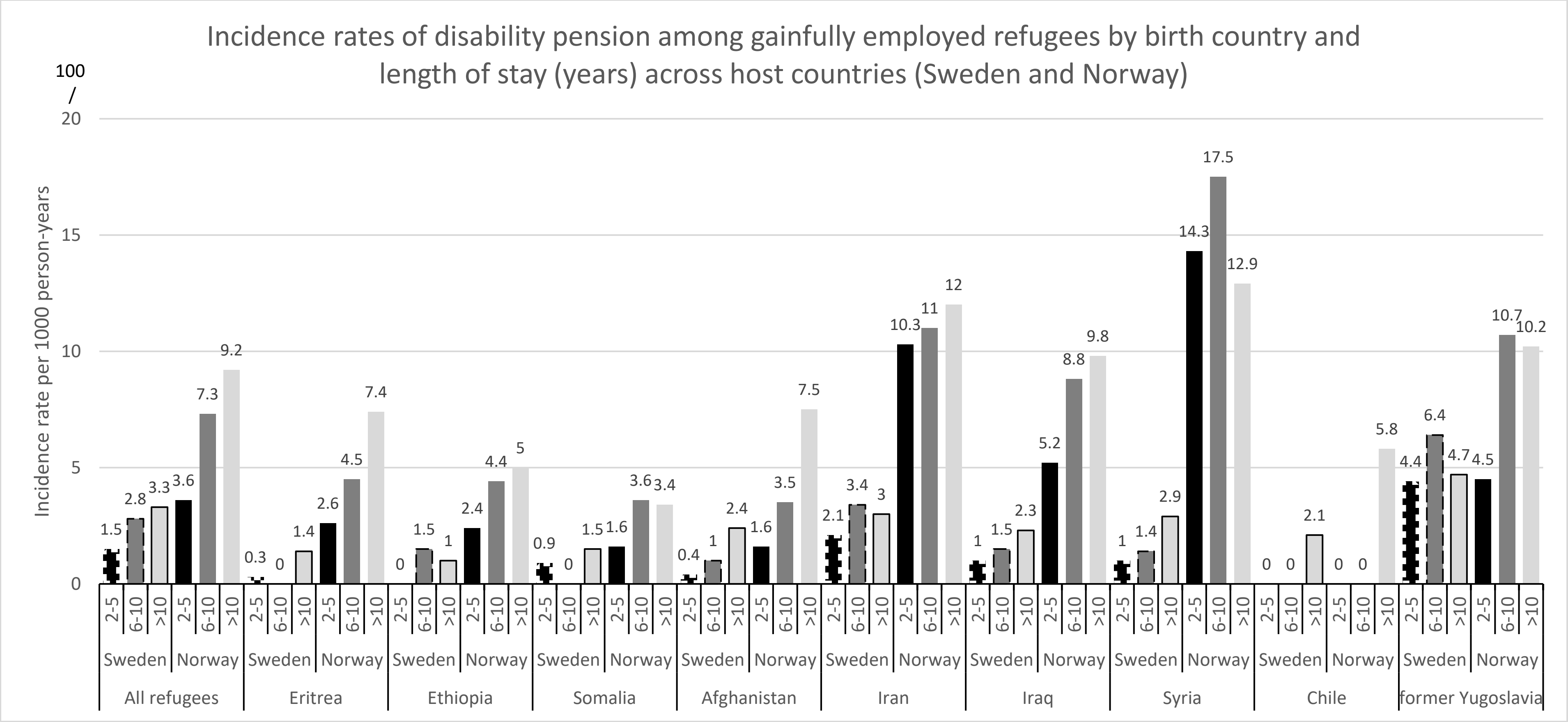

Supplement: Supplementary material [file SJWEH-50-279-S001.pdf]
